# High albumin level is a predictor of favorable response to immunotherapy in autoimmune encephalitis

**DOI:** 10.1038/s41598-018-19490-z

**Published:** 2018-01-17

**Authors:** Yoonhyuk Jang, Soon-Tae Lee, Tae-Joon Kim, Jin-Sun Jun, Jangsup Moon, Keun-Hwa Jung, Kyung-Il Park, Kon Chu, Sang Kun Lee

**Affiliations:** 1 Department of Neurology, Seoul National University Hospital, Seoul, South Korea; 2 Department of Neurology, Seoul National University Healthcare System Gangnam Center, Seoul, South Korea

## Abstract

There is no known biomarker that predicts the response to immune therapy in autoimmune synaptic encephalitis. Thus, we investigated serum albumin as a prognostic biomarker of early immune therapies in patients with autoimmune encephalitis. We enrolled patients who were diagnosed with definite autoimmune encephalitis and underwent IVIg treatment at Seoul National University Hospital from 2012 to 2017. Patients were dichotomized according to serum albumin prior to IVIg administration with a cut-off level of 4.0 g/dL, which was the median value of 50% of patients. Seventeen (53.1%) of the 32 patients with definite autoimmune encephalitis who received IVIg treatment in our hospital had low serum albumin (<4.0 g/dL). The initial disease severity (mRS ≥ 4) was the sole factor that predicted low albumin in autoimmune encephalitis patients using multivariate analysis (P = 0.013). The low albumin group exhibited a worse response to immune therapy at the third and fourth weeks from IVIg administration (P < 0.01 and P = 0.012, respectively), and recovery to activities of daily life without assistance was faster in the high albumin group (log-rank test for trend, P < 0.01). Our study found that pretreatment low serum albumin was a significant indicator of autoimmune encephalitis prognosis in the short-term and long-term.

## Introduction

Autoimmune encephalitis is a subacute onset encephalitis that occurs via autoimmune processes^1^. The most common forms of autoimmune encephalitis are anti-N-methyl D-aspartate receptor (NMDAR) and anti-leucine-rich glioma inactivated 1 (LGI1) encephalitis^2,3^. Treatments are immune therapies, including first-line drugs [e.g., immunoglobulin (IVIg) and steroids] and second-line drugs [e.g., cyclophosphamide, rituximab, and tocilizumab]^4–6^. The overall prognosis of autoimmune encephalitis largely depends on the classes of autoimmune antibodies, and a favorable initial response to immune therapies, such as IVIg and rituximab, results in better outcomes^4^.

However, no biomarkers are known to predict the response to immune therapy. Clinicians generally use their personal experience to judge whether to wait for treatment responses to first-line immune therapy or immediately move to much aggressive immune therapies. However, the earlier use of rituximab is associated with better outcome, and second-line immune therapies incur high costs and greater chances of infections; decisions to undergo the next step treatment must be timely and appropriate^4,7–9^.

Serum albumin is a well-known prognostic factor in various medical conditions^10–14^. Low albumin in neurological diseases is associated with poor prognosis in Guillain-Barre syndrome (GBS) treated with IVIg^15^. The half-life of immunoglobulin G (IgG) and albumin is prolonged up to 4 weeks by neonatal Fc receptor (FcRn) expression in endosomes^16^. We hypothesized that serum albumin would indirectly represent the recycling capacity of FcRn as a target of IVIg that IVIg would be less effective at inhibiting FcRn recycling in patients with low albumin^15^. We investigated whether serum albumin was associated with the treatment response of early immune therapies, including IVIg and rituximab, in patients with autoimmune encephalitis.

## Method

### Patient enrollment

We enrolled patients who were diagnosed with definite autoimmune encephalitis who underwent IVIg treatment at Seoul National University Hospital from 2012 to 2017. Definite autoimmune encephalitis was defined following the criteria from the recent publication^1^. Patients who initiated IVIg treatment in other faculties were excluded. Autoimmune antibodies in patient serum and cerebrospinal fluid (CSF) were confirmed in the three methods, as we described previously: immunostaining of rat brains to detect the presence of brain-reactive autoimmune antibodies, immunoblotting, and a cell-based immunochemistry kit (Euroimmun AG, Lübeck, Germany)^17,18^. We only analyzed adult patients who had all the laboratory tests measured within three days prior to IVIg administration to reflect the accurate status of patients with autoimmune encephalitis at the pretreatment level. We evaluated serum at four additional time points in some patients: the first, second, fourth, and eighth weeks after the first day of IVIg administration. The Institutional Review Board of the Seoul National University approved the Autoimmune Encephalitis Cohort Study (2520140040). We received written informed consent from all patients who were registered in the cohort, and all methods were performed in accordance with the relevant guidelines and regulations.

### Patient profiles and laboratory test measurement

Body weights and heights of all patients were measured prior to IVIg administration. Body mass index (BMI, kg/m^2^) was classified into three categories: low (<18.5), normal (18.5–24.9) and high (≥25). Autoimmune synaptic encephalitis was categorized into three groups: anti-NMDAR antibody, anti-LGI1 antibody, and others, including anti-γ-aminobutyric acid B (GABA-B) antibody, anti-contactin-associated protein-like 2 (Caspr2) antibody, and anti-α-amino-3-hydroxy-5-methyl-4-isoxazolepropionic acid 2 (AMPA2) antibody.

Serum albumin was analyzed using the bromocresolgreen method in an automatic clinical chemistry analyzer (Roche Hitachi Modular; Roche Diagnostics Corp. Indianapolis, IN, USA). The reference range of albumin was set to 3.3 g/dL to 5.2 g/dL. The glomerular filtration rate (GFR) was calculated using the Modification of Diet in Renal Disease equation^19^. Calculated globulin (CG) was derived from the difference between total protein and serum albumin. ΔAlbumin and ΔCG were calculated between pretreatment and the first week after IVIg.

### Profile of treatment and the initial response to immune therapies

All the patients with autoimmune encephalitis received first-line immune therapies as soon as possible after diagnosis. The first-line immune therapy was IVIg administration (0.4 g/kg of IVIg for 5 days) alone or in combination with a corticosteroid pulse (500–1000 mg of methylprednisolone for 5 days) or plasma exchange (4 or more). Second-line immune therapy was rituximab (375 mg/m^2^), tocilizumab (8 mg/kg), or cyclophosphamide. Third-line immune therapies, including proleukin, bortezomib, and interferon, were considered for patients who did not respond well to first- and second-line treatments.

An initial response to immune therapy was acknowledged if the modified Rankin Scale (mRS) of the patients improved within one month after the first day of IVIg administration^20^. A good long-term outcome was defined as recovery to mRS ≤ 2, which indicates that activities of daily life were possible without assistance during a follow-up of 2 years^4^.

### Statistical analysis

Patients were dichotomized according to serum albumin prior to IVIg administration with a cut-off level of 4.0 g/dL, which was the median value of 50% of patients.

Categorical data are summarized as percentages, and continuous data are represented as the means ± standard deviation (SD) for normally distributed variables and medians [interquartile range (IQR)] for non-normally distributed variables.

Fisher’s exact test was used for binary and higher orders of categorical variables. Continuous variables were evaluated using the Mann-Whitney U test. Multivariate logistic regression was performed to identify the significant factors associated with low serum albumin and initial responses to immune therapy. A Kaplan-Meier graph was constructed to evaluate the activity of daily life without assistance, which was defined as mRS ≤ 2. GraphPad Prism, version 6.0 for Mac (GraphPad Software, CA, USA), and STATA 14 (StataCorp LLC., Texas, USA) were used for analyses, and a P-value < 0.05 was considered statistically significant.

### Data availability statement

All data generated or analyzed during this study are included in this published article or in the supplementary information.

### Ethical Publication Statement

We confirm that we have read the journal’s position on the issues involved in ethical publication and affirm that this report is consistent with these guidelines.

## Results

### Factors associated with low serum albumin

Thirty-four of the 66 patients enrolled were excluded: eight patients were under 13 years old and exhibited different normal ranges of laboratory tests; 20 patients had incomplete studies, and six patients did not have laboratory tests within 72 hours prior to IVIg treatment. Seventeen (53.1%) of the 32 patients with definite autoimmune encephalitis who received IVIg treatment in our hospital exhibited low serum albumin (<4.0 g/dL) (Table 1). The type of autoimmune antibodies was significantly associated with serum albumin (P = 0.042). Anti-NMDAR encephalitis was frequent in the low albumin group (11, 64.7%), and anti-LG1 encephalitis was frequent in the high albumin group (10, 66.7%). Laboratory tests of liver and kidney functions, which affect albumin half-life in serum, were compared between the two groups. Aspartate aminotransferase (AST) and alanine aminotransferase (ALT) were higher in patients with low albumin (P < 0.01 and P = 0.037, respectively). Serum creatinine was lower in patients with low albumin (P = 0.049), and GFR was not significantly different between the two groups (P = 0.104). C-reactive protein elevation was more frequent in patients with low albumin (P = 0.041). The duration from onset of the symptoms to measurement of serum albumin was not significant (P = 0.179), but the low albumin group suffered more severe symptoms at initial presentation (P < 0.01).

**Table 1:** Factors associated with low albumin level.

		Total (N = 32)	Low albumin (<4.0) (n = 17)	High albumin (≥4.0) (n = 15)	P-value
**Demographics and Disease**					
Age (years)		47.1 ± 20.7	44.5 ± 22.5	50.1 ± 18.9	0.570*
Male (% of cases)		16 (50.0%)	6 (35.3%)	10 (66.7%)	0.156
BMI (% of cases)	Low (<18.5 kg/m^2^)	4 (12.5%)	4 (23.5%)	0	0.121
	Normal (18.5 to 24.9 kg/m^2^)	19 (59.4%)	10 (58.8%)	9 (60.0%)	
	High (≥25 kg/m^2^)	9 (28.1%)	3 (17.7%)	6 (40.0%)	
Autoantibody (% of cases)	Anti-NMDAR	15 (46.9%)	11 (64.7%)	4 (26.7%)	0.042
	Anti-LGI1	14 (43.8%)	4 (23.5%)	10 (66.7%)	
	Others (Anti-GABAB, Caspr2, AMPA2)	3 (9.4%)	2 (11.8%)	1 (6.7%)	
Tumor (% of cases)		9 (28.1%)	6 (35.3%)	3 (20.0%)	0.444
**Laboratory**					
Total cholesterol (mg/dL)		155.5 ± 34.3	145.2 ± 30.2	167.1 ± 35.9	0.124*
AST (U/L)		34.6 ± 33.2	48.0 ± 41.0	19.5 ± 7.8	0.0003*
ALT (U/L)		38.3 ± 38.0	50.7 ± 48.6	24.1 ± 9.8	0.037*
ALP (U/L)		67.1 ± 34.1	71.5 ± 41.4	62.1 ± 23.8	0.806*
Total bilirubin (mg/dL)		0.66 ± 0.33	0.56 ± 0.22	0.77 ± 0.40	0.120*
Creatinine Kinase (U/L)^†^		124 [63–227]	129 [62–718]	124 [63–227]	0.922*
C-reactive protein elevation (>3 mg/L)		17 (53.1%)	7 (87.5%)	1 (12.5%)	0.041
Serum sodium (mmoL/L)		137.4 ± 6.9	138.3 ± 8.2	136.4 ± 5.1	0.183*
Blood urea nitrogen (mg/dL)		14.0 ± 6.9	15.0 ± 8.2	12.9 ± 5.2	0.650*
Serum creatinine (mg/dL)		0.73 ± 0.21	0.66 ± 0.20	0.80 ± 0.20	0.049*
GFR (ml/min)		101.1 [88.5–>120]	>120 [87.6–>120]	94 [88.5–115.3]	0.104*
CSF (% of cases)^‡^	Lymphocytosis (WBC > 5)	15 (55.6%)	11 (73.3%)	4 (33.3%)	0.057
	Elevated protein (>45 g/dl)	13 (48.2%)	6 (40.0%)	7 (58.3%)	0.449
EEG abnormality (% of cases)		11 (34.4%)	5 (29.4%)	6 (40.0%)	0.721
Brain MRI abnormality (% of cases)		13 (40.6%)	7 (41.2%)	6 (40.0%)	1.000
**Duration**					
Onset to Albumin (weeks)		5.25 [3–14]	5 [2–8.5]	5 [2.5–16]	0.179*
Onset to first-line IT (weeks)		5 [3–12]	4 [2–10.5]	6 [4–24]	0.125*
**Prognosis**					
ICU admission		9 (28.1%)	7 (41.2%)	2 (13.3%)	0.122
ICU duration (days)^§^		34.5 [21.5–60]	34.5 [23–60]	35 [10–60]	0.613*
Mechanical Ventilator		8 (25.0%)	6 (35.3%)	2 (13.3%)	0.229
Initial Severity (mRS ≥ 4) (% of cases)		17 (53.1%)	12 (85.7%)	2 (14.3%)	0.002
Response to initial immune therapies (% of cases)	Second week	15 (46.9%)	5 (29.4%)	10 (66.7%)	0.074
	Third week	18 (56.3%)	5 (29.4%)	13 (86.7%)	0.002
	Fourth week	20 (62.5%)	7 (41.2%)	13 (86.7%)	0.012

BMI = body mass index; NMDAR = N-methyl D-aspartate receptor; LGI1 = leucine-rich glioma inactivated 1; GABAB = γ-aminobutyric acid B; Caspr2 = contactin-associated protein-like 2; AMAPA2 = α-amino-3-hydroxy-5-methyl-4-isoxazolepropionic acid 2; AST = aspartate aminotransferase; ALT = alanine aminotransferase; ALP = Alkaline phosphatase; GFR = glomerular filtration rate; CSF = cerebrospinal fluid; WBC = white blood cell; EEG = electroencephalogram; MRI = magnetic resonance imaging; ICU = intensive care unit; mRS = modified Rankin Scale; Fisher’s exact test unless otherwise stated; *Mann-Whitney U test; ^†^17 patients were analyzed because the data of 15 patients were missing; ^‡^27 patients were analyzed because the data of 5 patients were missing; ^§^Eight patients were analyzed because the admission date of one patient was ambiguous.

Multivariate analysis demonstrated that the initial disease severity (mRS ≥ 4) was the sole factor that predicted low albumin in autoimmune encephalitis patients (P = 0.013) (Supplemental Table 1).

### Low serum albumin predicts poor outcomes

Patients with low albumin had a worse response to immune therapy at the third and fourth weeks after IVIg administration. Responders were 5 (29.4%) in the low albumin group vs. 10 (66.7%) in the high albumin group at the second week, 5 (29.4%) vs. 13 (86.7%) at the third week, and 7 (41.2%) vs. 13 (86.7%) at the fourth week after treatment (Table 1, P = 0.074, P < 0.01, and P = 0.012, respectively). Therefore, we evaluated which clinical factors could predict the initial response to immune therapy within one month (Table 2). Notably, the initial disease severity was irrelevant with the early response to immune therapy (P = 0.087 for the second week, P = 0.283 for the third week, P = 0.068 for the fourth week). At the second week from first-line immune therapy, sex, the type of autoimmune antibody, and CSF lymphocytosis were significant in univariate analysis but not in multivariate analysis. In contrast, only low albumin was a significant factor in the prediction of poor responders to immune therapy at the third and fourth week, even in multivariate analyses (Table 3, P < 0.01 and P = 0.034, respectively). The administration of first-line and second-line immune therapies was not faster or biased for the good responders. Rather, the second-line immune therapy rituximab tended to be injected more frequently for the poor responders at the second week (P = 0.013).

**Table 2:** Factors associated with prognosis.

	Initial response to immune therapy										
		All cases (N = 32)	Second week			Third week			Fourth week		
			Responder (n = 15)	Non-responder (n = 17)	P-value	Responder (n = 18)	Non-responder (n = 14)	P-value	Responder (n = 20)	Non-responder (n = 12)	P-value
**Demographics and Disease**											
Age (<50) (% of cases)		15 (46.9%)	5 (33.3%)	10 (58.8%)	0.178	8 (44.4%)	7 (50.0%)	1.000	9 (45.0%)	6 (50.0%)	1.000
Male (% of cases)		16 (50.0%)	12 (80.0%)	4 (23.5%)	0.004	12 (66.7%)	4 (28.6%)	0.073	12 (60.0%)	4 (33.3%)	0.273
Autoantibody (% of cases)	Anti-NMDAR	15 (46.9%)	3 (20.0%)	12 (70.6%)	0.010	6 (33.3%)	9 (64.3%)	0.256	7 (35.0%)	8 (66.7%)	0.229
	Anti-LGI1	14 (43.8%)	10 (66.7%)	4 (23.5%)		10 (55.6%)	4 (28.6%)		11 (55.0%)	3 (25.0%)	
	Others (Anti-GABAB, Caspr2, AMPA2)	3 (9.4%)	2 (13.3%)	1 (5.9%)		2 (11.1%)	1 (7.1%)		2 (10.0%)	1 (8.3%)	
Tumor (% of cases)		9 (28.1%)	2 (13.3%)	7 (41.2%)	0.122	4 (22.2%)	5 (35.7%)	0.453	5 (25.0%)	4 (33.3%)	0.696
BMI (% of cases)	Low (<18.5 kg/m^2^)	4 (12.5%)	1 (6.7%)	3 (17.7%)	0.776	1 (5.6%)	3 (21.4%)	0.454	2 (10.0%)	2 (16.7%)	1.000
	Normal (18.5 to 24.9 kg/m^2^)	19 (59.4%)	9 (60.0%)	10 (58.8%)		12 (66.7%)	7 (50.0%)		12 (60.0%)	7 (58.3%)	
	High (≥25 kg/m^2^)	9 (28.2%)	5 (33.3%)	4 (23.5%)		5 (27.8%)	4 (28.6%)		3 (25.0%)	6 (30.0%)	
Initial Severity (mRS ≥ 4) (% of cases)		14 (43.8%)	4 (26.7%)	10 (58.8%)	0.087	6 (33.3%)	8 (57.1%)	0.283	6 (30.0%)	8 (66.7%)	0.068
**Laboratory**											
Low albumin level (<4.0) (% of cases)		17 (53.1%)	5 (33.3%)	12 (70.1%)	0.074	5 (27.8%)	12 (85.7%)	0.002	7 (35.0%)	10 (83.3%)	0.012
Total cholesterol level (mean, mg/dL)		155.5 (34.3)	159.1 (34.7)	152.4 (34.7)	0.734*	159.8 (31.9)	150 (37.8)	0.436*	159.8 (30.9)	148.3 (39.8)	0.360*
LFT abnormality: AST > 40 or ALT > 40 (% of cases)		9 (28.1%)	2 (13.3%)	7 (41.2%)	0.122	3 (16.7%)	6 (42.9%)	0.132	3 (15.0%)	6 (50.0%)	0.049
C-reactive protein elevation (>3 mg/L)		8 (25.0%)	3 (20.0%)	5 (29.4%)	0.691	3 (16.7%)	5 (35.7%)	0.252	3 (15.0%)	5 (41.7%)	0.116
Creatinine Kinase (U/L)^†^		289.7 ± 366.9	242.3 ± 2276.5	315.5 ± 418.5	0.763*	389.6 ± 464.2	219.8 ± 287.3	0.283*	348.6 ± 445.1	237.3 ± 299.0	0.563*
GFR (ml/min)		113.6 ± 39.8	100.7 ± 26.1	125.1 ± 46.6	0.117*	103.6 ± 25.8	126.5 ± 50.8	0.210*	106.3 ± 31.2	125.8 ± 50.2	0.242*
CSF (% of cases)	Lymphocytosis (WBC > 5)	15 (55.6%)	3 (25.0%)	12 (80.0%)	0.007	6 (40.0%)	9 (75.0%)	0.121	7 (41.2%)	8 (80.0%)	0.107
	Elevated protein (>45 g/dl)	13 (48.2)	6 (50.0%)	7 (46.7%)	1.000	7 (46.7%)	6 (50.0%)	1.000	8 (47.1%)	5 (50.0%)	1.000
EEG abnormality (% of cases)		11 (34.4%)	5 (33.3%)	6 (35.3%)	1.000	6 (33.3%)	5 (35.7%)	1.000	7 (35.0%)	4 (33.3%)	1.000
Brain MRI abnormality (% of cases)		13 (40.6%)	9 (60.0%)	4 (23.5%)	0.070	10 (55.6%)	3 (21.4%)	0.075	10 (50.0%)	3 (25.0%)	0.267
**Management**											
Onset to first-line immune therapy (weeks)		5 [3–12]	5.5 [4–32]	4 [2–10.5]	0.123*	4.5 [3–24]	6.5 [2–11]	0.568*	5 [3.5–20]	5 [2–9.3]	0.212*
Combination with steroid pulse		21 (65.6%)	11 (73.3%)	10 (58.8%)	0.472	13 (72.2%)	8 (57.1%)	0.465	15 (75.0%)	6 (50.0%)	0.250
Administration of second-line immune therapy		24 (75.0%)	8 (53.3%)	16 (94.1%)	0.013	11 (61.1%)	13 (92.9%)	0.053	13 (65.0%)	11 (91.7%)	0.204
Duration to second-line immune therapy from first-line (days)		6 [5–13]	7 [5.5–19.5]	6 [4.5–10.5]	0.579*	8 [6–13]	6 [4–8]	0.325*	8 [6–13]	5 [3–8]	0.130*

NMDAR = N-methyl D-aspartate receptor; LGI1 = leucine-rich glioma inactivated 1; GABAB = γ-aminobutyric acid B; Caspr2 = contactin-associated protein-like 2; AMAPA2 = α-amino-3-hydroxy-5-methyl-4-isoxazolepropionic acid 2; BMI = body mass index; mRS = modified Rankin Scale; LFT = liver function tests; AST = aspartate aminotransferase; ALT = alanine aminotransferase; GFR = glomerular filtration rate; CSF = cerebrospinal fluid; WBC = white blood cell; EEG = electroencephalogram; MRI = magnetic resonance imaging; Fisher’s exact test unless otherwise stated; *Mann-Whitney U test; ^†^17 patients were analyzed because the data of 15 patients were missing. ^‡^27 patients were analyzed because the data of 5 patients were missing.

**Table 3:** Multivariate analysis associated with response to immune therapy.

	Second week		Third week		Fourth week	
	OR (95% CI)	P-value	OR (95% CI)	P-value	OR (95% CI)	P-value
Male	0.14 (0.02–1.21)	0.073				
Autoantibody	1.28 (0.24–6.88)	0.773				
LFT abnormality					0.26 (0.04–1.62)	0.149
CSF lymphocytosis	0.13 (0.02–1.00)	0.050				
Albumin < 4.0			0.06 (0.01–0.39)	0.003	0.13 (0.02–0.86)	0.034

**Figure 1:**
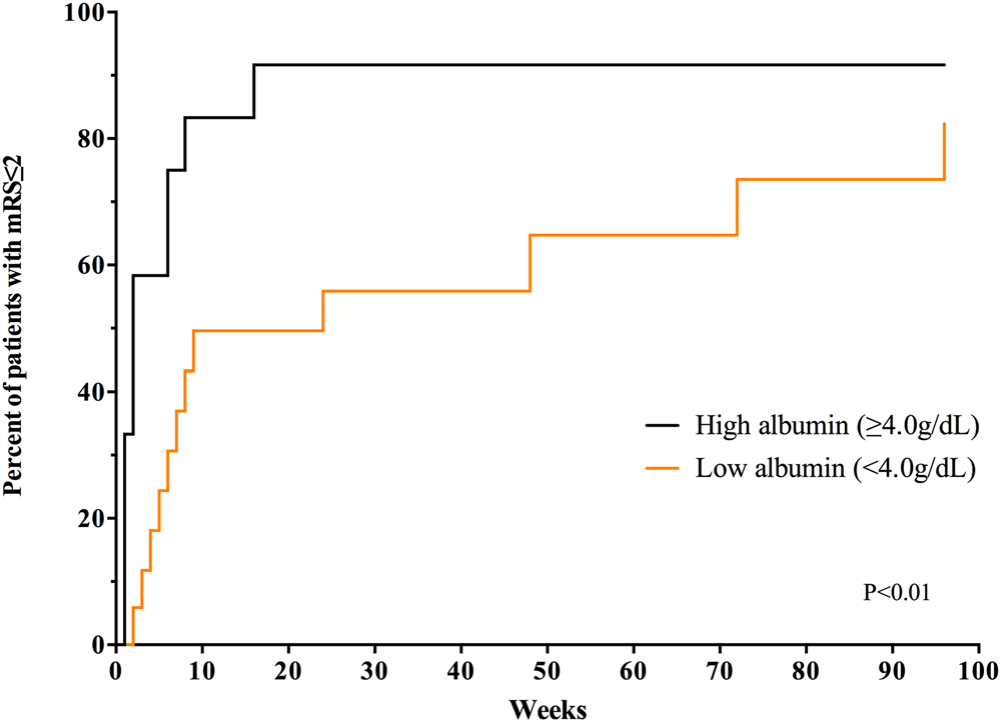
Long-term outcome according to pretreatment serum albumin in patients with autoimmune encephalitis. Kaplan-Meier analysis shows that patients with low albumin (<4.0 g/dL) exhibit significantly delayed recovery to activities of daily life without assistance (modified Rankin Scale ≤ 2) (P < 0.01).

We compared long-term outcomes between the two groups. Recovery to mRS ≤ 2, which indicates that activities of daily life are possible without assistance, was faster in the high albumin group than the low albumin group during a follow-up of 2 years. (log-rank test for trend, P < 0.01) (Fig. 1). Rituximab was more frequently administered to patients with low albumin (Supplemental Table 2, weekly P = 0.013, monthly P = 0.031).

### Trends in albumin levels after IVIg

**Figure 2:**
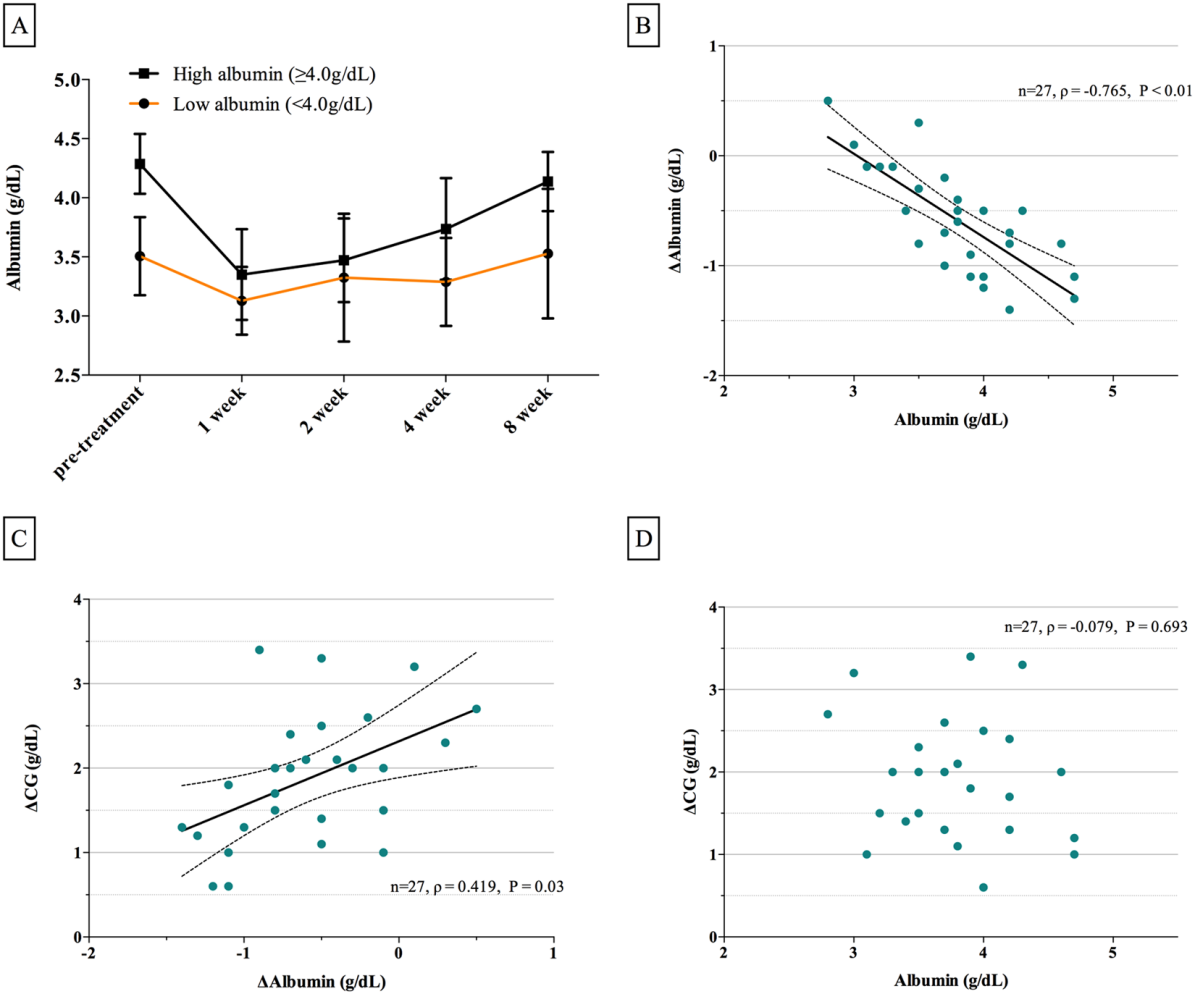
The association between albumin and calculated globulin (CG) in patients with autoimmune encephalitis. (**A**) Serial changes in serum albumin before and over four time points (the first, second, fourth, and eighth weeks) after IVIg. Serum albumin changed less after IVIg in patients with low albumin (<4.0 g/dL) than patients with high albumin (≥4.0 g/dL). Dots indicate medians, and vertical bars indicate interquartile range. (**B**) Scatterplot displaying the correlation between albumin and ΔAlbumin (pretreatment albumin - albumin at the first week after IVIg). (**C**) Scatterplot displaying the correlation between ΔAlbumin and ΔCG (CG at the first week after IVIg - pretreatment CG) (**D**) Scatterplot displaying the correlation between albumin and ΔCG.

We evaluated serial serum albumin and its changes between time intervals in autoimmune encephalitis patients (Fig. 2(A), Supplemental Tables 3 and 4). Albumin levels in the low albumin group dropped significantly less the first week after IVIg administration than in the high albumin group (P < 0.01). Both groups gradually recovered serum albumin to initial levels after the first week, and albumin levels were significantly different the fourth week after IVIg. However, the speed of recovery was not significantly different between the two groups.

The association between serum albumin and CG (total protein-globulin) was investigated. Pretreatment serum albumin inversely correlated to ΔAlbumin (pretreatment albumin - albumin at the first week after IVIg) (Fig. 2(B), ρ = −0.765, P < 0.01), and ΔAlbumin was proportional to ΔCG (CG at the first week after IVIg - pretreatment CG) (Fig. 2(C), ρ = 0.419, P = 0.03). However, pretreatment serum albumin showed no significant correlation to ΔCG (Fig. 2(D), P = 0.693).

## Discussion

We demonstrated that autoimmune encephalitis patients with low serum albumin (<4.0 g/dL) exhibited poor initial response to immune therapies at the third and fourth weeks after IVIg treatment compared to the high albumin patients. Low albumin was associated with initial disease severity (mRS ≥ 4), but neither the initial severity nor the type of autoimmune antibody predicted the early response to immune therapies. Long-term outcome was also poor in the low albumin group, and the mRS improved more slowly than in the high albumin group. Patients with low albumin had smaller fluctuations in albumin levels after IVIg, and ΔAlbumin was inversely correlated with pretreatment serum albumin. However, ΔCG was not associated with pretreatment serum albumin.

This report shows that initial serum albumin may be a prognostic indicator in autoimmune encephalitis patients. The predictive value of albumin level was confirmed in various diseases, including patients with heart failure and autoimmune diseases, such as Kawasaki disease and GBS^12–15^. Serum albumin in autoimmune encephalitis strongly correlated with the initial patient presentation. Previous studies suggested that this observation may be due to the hyperactive catabolic state of critically ill patients^10,21^. AST and ALT represent liver dysfunction, and the levels of these enzymes were significantly higher; C-reactive protein is a biomarker of inflammation, and it was more elevated. Serum creatinine, which indicates total muscle mass, was lower in the low albumin group. Moreover, BMI did not correlate with the low albumin in autoimmune encephalitis, as observed in patients with heart failure^12,13^. However, the mechanism of the hypoalbuminemia may not be identical to heart failure patients. Serum sodium level and GFR, which influence albumin loss^13,22,23^, were not associated with albumin level in autoimmune encephalitis.

We revealed a correlation between albumin and CG after IVIg treatment. Our results demonstrated that the reduction in albumin level after IVIg was significantly smaller in patients with low albumin (Fig. 2(A,B)). The reduction in serum albumin after IVIg was interpreted as exhaustion of the recycling pathway via FcRn^15^. Higher pretreatment albumin may increase FcRn as the target for IVIg and result in a good response.

However, our results do not limit the therapeutic effect of IVIg to saturation of the FcRn in autoimmune encephalitis. Pretreatment albumin was not associated with ΔCG, rather than it showing inverse association with ΔCG (Fig. 2(D)). This result does not conflict with a previous study that reported that smaller fluctuations in serum IgG after IVIg were significantly associated with a poor prognosis in patients with GBS^24^.

Our data should be cautiously interpreted and reproduced in more patients with autoimmune encephalitis. First, there is a possibility that the pharmacokinetics of IVIg are different between GBS and autoimmune encephalitis. Second, previous studies in GBS investigated serum IgG, but we analyzed CG. Third, the initial response to immune therapies was not the sole effect of IVIg because patients received second-line rituximab treatment within one month. We do not know the synergy between IVIg and rituximab, but the earlier use of rituximab treatment is associated with better outcomes. However, our results demonstrated that second-line immune therapies were performed less often in the responders to immune therapies compared to non-responders, suggesting that the initial response to treatment within a month might be largely attributed to IVIg. The half-life of rituximab, which is also IgG, may be longer in the high albumin group, which exhibited better recycling capacity of FcRn, and this capacity contributed a good prognosis.

Further studies are needed to validate the clinical implication of serum albumin in the treatment of autoimmune encephalitis. For example, research on whether the protocol should be subdivided according to pretreatment albumin levels during treatment with IVIg or whether albumin may be used as a reference during the first-line early rituximab trial must be examined.

In summary, our study found that low pretreatment serum albumin was a significant indicator of the short-term and long-term prognosis of autoimmune encephalitis. This association may be helpful in the clinic to predict poor outcomes and the initial response to immune therapies. These results should be used as a reference for further studies on the early start of additional therapies.

## Electronic supplementary material

Supplemental tables
