# Supplementary material for: High albumin level is a predictor of favorable response to immunotherapy in autoimmune encephalitis
Source: Sci Rep. 2018 Jan 17;8:1012. doi: 10.1038/s41598-018-19490-z (PMC5772466; doi:10.1038/s41598-018-19490-z)
Supplement: Supplementary file 1 — Supplemental tables [file 41598_2018_19490_MOESM1_ESM.doc]

**High albumin level is a predictor of favorable response to immunotherapy in autoimmune encephalitis**

Yoonhyuk Jang, MD^1, 2, ¶^, Soon-Tae Lee, MD, PhD^1, ¶^, Tae-Joon Kim, MD^1^, Jin-Sun Jun, MD^1^, Jangsup Moon, MD, PhD^1^, Keun-Hwa Jung, MD, PhD^1^, Kyung-Il Park, MD, PhD^2^, Kon Chu, MD, PhD^1^, Sang Kun Lee, MD, PhD^1^

*^1^Department of Neurology, Seoul National University Hospital, Seoul, South Korea.*

*^2^Department of Neurology, Seoul National University Healthcare System Gangnam Center, Seoul, South Korea*

*^¶^ These two authors contributed equally to this work.*

**Correspondence to:**

Kon Chu, MD, PhD.

Department of Neurology, Seoul National University Hospital

101, Daehak-ro, Jongno-gu, Seoul 03080, South Korea

Tel.: +82-2-2072-1878; Fax: +82-2-2072-7424; E-mail: [stemcell.snu@gmail.com](mailto:stemcell.snu@gmail.com)

Or

Sang Kun Lee, MD, PhD.

Department of Neurology, Seoul National University Hospital

101, Daehak-ro, Jongno-gu, Seoul 03080, South Korea

Tel.: +82-2-2072-2923; Fax: +82-2-3672-7553; E-mail: [sangkun2923@gmail.com](mailto:sangkun2923@gmail.com)

| **Supplemental Table 1. Multivariate analysis associated with low albumin** | | | |
| --- | --- | --- | --- |
|  | Univariate | Multivariate | |
|  | P-value | OR (95% CI) | P-value |
| Autoantibody | 0.042 | 1.70 (0.27-10.72) | 0.573 |
| LFT abnormality: AST >40 or ALT >40 | 0.122 | 1.35 (0.12-14.60) | 0.807 |
| C-reactive protein elevation (>3mg/L) | 0.041 | 2.71 (0.17-44.51) | 0.484 |
| Initial Severity (mRS≥4) | 0.002 | 15.44 (1.19-200.23) | 0.036 |
| LFT=liver function test | | | |

| **Supplemental Table 2. Profile of immune therapy for patients with autoimmune encephalitis** | | | | |
| --- | --- | --- | --- | --- |
| Immune therapy (% of cases) | Total (N=32) | Low albumin (<4.0) (n=17) | High albumin (≥4.0) (n=15) | P-value |
| IVIg | 32 (100%) | 17 (100%) | 15 (100%) | 1.000 |
| Steroid pulse | 21 (65.6%) | 11 (64.7%) | 10 (66.7%) | 1.000 |
| Weekly rituximab | 24 (75.0%) | 16 (94.1%) | 8 (53.3%) | 0.013 |
| Monthly rituximab | 18 (56.3%) | 13 (76.5%) | 5 (33.3%) | 0.031 |
| Tocilizumab | 15 (46.9%) | 10 (58.8%) | 5 (33.3%) | 0.178 |
| Others | 5 (15.6%) | 4 (23.5%) | 1 (6.7%) | 0.338 |
| Others include proleukin, bortezomib, and cyclophoaphamide; IVIg=immunoglobulin | | | | |

| **Supplemental Table 3. Albumin level at five time points** | | | | | | |
| --- | --- | --- | --- | --- | --- | --- |
|  | Albumin | Before IVIg | After IVIg | | | |
|  |  |  | 1^st^ week | 2^nd^ week | 4^th^ week | 8^th^ week |
| High albumin (≥4.0g/dL) | Number | 15 | 10 | 7 | 11 | 8 |
|  | Median [IQR] | 4.2 [4.0-4.5] | 3.45 [2.9-3.6] | 3.4 [3.2-3.8] | 3.8 [3.2-4.0] | 4.15 [4.0-4.3] |
|  | Mean±SD | 4.29±0.25 | 3.35±0.38 | 3.47±0.35 | 3.74±0.43 | 4.14±0.25 |
| Low albumin (<4.0g/dL) | Number | 17 | 17 | 16 | 17 | 11 |
|  | Median [IQR] | 3.5 [3.3-3.8] | 3.1 [3.0-3.3] | 3.2 [2.9-3.65] | 3.2 [3.1-3.5] | 3.6 [3.2-3.9] |
|  | Mean±SD | 3.51±0.33 | 3.13±0.08 | 3.32±0.54 | 3.29±0.37 | 3.53±0.55 |
| P-value | | | 0.101 | 0.313 | 0.009 | 0.009 |
| IVIg= immunoglobulin; before IVIg=before the administration of IVIg; After IVIg=time after the administration of IVIg; number=number of the patients who was investigated at the indicated time points; IQR=interquartile range; SD=standard deviations; | | | | | | |

| **Supplemental Table 4. ΔAlbumin at four time intervals** | | | | | |
| --- | --- | --- | --- | --- | --- |
| ΔAlbumin (g/dL) | | Pre-1week | 2week-1week | 4week-1week | 8week-1week |
| High albumin (≥4.0g/dL) | Median [IQR] | -0.95 [-1.2--0.7] | +0.3 [0-0.4] | +0.4 [0.3-0.5] | +0.95 [0.3-1.3] |
|  | Mean | -0.94±0.32 | +0.23±0.41 | +0.37±0.45 | +0.87±0.57 |
| Low albumin (<4.0g/dL) | Median [IQR] | -0.4 [-0.7--0.1] | +0.05 [-0.15-0.4] | +0.1 [-0.1-0.4] | +0.4 [0.2-0.8] |
|  | Mean | -0.38±0.45 | +0.17±0.53 | +0.16±0.37 | +0.42±0.51 |
| P-value | | 0.003 | 0.546 | 0.175 | 0.227 |
| ΔAlbumin=pretreatment albumin-albumin at the first week after IVIg | | | | | |
